# Beneficial Effects of Ethanolic and Hexanic Rice Bran Extract on Mitochondrial Function in PC12 Cells and the Search for Bioactive Components

**DOI:** 10.3390/molecules200916524

**Published:** 2015-09-11

**Authors:** Stephanie Hagl, Dirk Berressem, Bastian Bruns, Nadine Sus, Jan Frank, Gunter P. Eckert

**Affiliations:** 1 Department of Pharmacology, Biocenter Campus Riedberg, Goethe-University of Frankfurt, Frankfurt 60438, Germany; E-Mails: dirk.berressem@nutritional-neuroscience.com (D.B.); s8132827@stud.uni-frankfurt.de (B.B.); gunter.eckert@nutritional-neuroscience.com (G.P.E.); 2 Institute of Biological Chemistry and Nutrition, University of Hohenheim, Stuttgart 70599, Germany; E-Mails: nadine.sus@nutres.de (N.S.); jan.frank@nutres.de (J.F.)

**Keywords:** mitochondria, rice bran extract, vitamin E, tocopherol, tocotrienol, PC12 cells, aging, prevention, neurodegeneration

## Abstract

Mitochondria are involved in the aging processes that ultimately lead to neurodegeneration and the development of Alzheimer’s disease (AD). A healthy lifestyle, including a diet rich in antioxidants and polyphenols, represents one strategy to protect the brain and to prevent neurodegeneration. We recently reported that a stabilized hexanic rice bran extract (RBE) rich in vitamin E and polyphenols (but unsuitable for human consumption) has beneficial effects on mitochondrial function *in vitro* and *in vivo* (doi:10.1016/j.phrs.2013.06.008, 10.3233/JAD-132084). To enable the use of RBE as food additive, a stabilized ethanolic extract has been produced. Here, we compare the vitamin E profiles of both extracts and their effects on mitochondrial function (ATP concentrations, mitochondrial membrane potential, mitochondrial respiration and mitochondrial biogenesis) in PC12 cells. We found that vitamin E contents and the effects of both RBE on mitochondrial function were similar. Furthermore, we aimed to identify components responsible for the mitochondria-protective effects of RBE, but could not achieve a conclusive result. α-Tocotrienol and possibly also γ-tocotrienol, α-tocopherol and δ-tocopherol might be involved, but hitherto unknown components of RBE or a synergistic effect of various components might also play a role in mediating RBE’s beneficial effects on mitochondrial function.

## 1. Introduction

Rising life expectancy leads to increased prevalence of age-related diseases like cancer, cardiovascular and neurodegenerative diseases [1]. Alzheimer’s disease (AD) is the most common neurodegenerative disorder and affects one out of four individuals over the age of 85 [2]. Current therapies available for AD patients only attenuate symptoms, but are not able to stop disease progression or to even cure the disease [3]. Accordingly prevention or very early intervention using pharmaceuticals or plant formulations might be a valuable approach for slowly progressing neurodegenerative diseases like AD [4].

Mitochondrial dysfunction has been found to occur very early in AD development and therefore plays a key role in disease progression [5,6]. Prevention of mitochondrial dysfunction might therefore be a promising target to prevent neurodegeneration and AD [7]. Recent research revealed that plant components such as polyphenols and vitamin E are suitable for the protection of brain mitochondrial and cognitive function [8,9,10]. Some reports suggest that plant extracts containing several of the above-mentioned substances are even more effective than their single components [11].

We have recently reported that a stabilized hexanic rice bran extract (RBE) containing high concentrations of vitamin E improved mitochondrial function in brain cells of young guinea pigs [12] and in a cell culture model of early AD [13] by increasing mitochondrial content and resistance of cells against nitrosative stress. Based on these positive results, it is our intention to develop RBE as food additive to create functional foods for the protection of brain cells against neurodegeneration. For this purpose, IT & M (Giza, Egypt) developed a stabilized ethanolic RBE that would be more suitable for human use than the hexanic extract.

The composition of bioactive compounds in the two extracts may differ due to the use of different extraction solvents. We therefore analyzed and compared the profiles of vitamin E compounds in the extracts as well as the effects of both extracts on mitochondrial function (ATP, mitochondrial membrane potential, mitochondrial respiration and mitochondrial biogenesis) in PC12 cells. Furthermore, we aimed to identify components responsible for the mitochondria-protective effects of RBE. For this purpose, we tested the effects of single substances as well as fractions of RBE on mitochondrial function in PC12 cells.

## 2. Results and Discussion

### 2.1. Vitamin E Concentrations

Concentrations of vitamin E congeners (tocopherols and tocotrienols) were determined in ethanolic and hexanic RBE. Total amounts of vitamin E were similar in both extracts (Table 1). γ-Tocotrienol was the most abundant vitamin E compound in both extracts and its concentrations were higher in the ethanolic RBE. In the hexanic RBE, δ-tocotrienol was the second most abundant vitamin E compound, followed by δ-, γ- and β-tocopherol. In ethanolic RBE, γ-tocopherol and δ-tocotrienol were the second and third most common congeners. β-Tocotrienol was not detectable in either extract (Table 1). Taken together, vitamin E concentrations in ethanolic and hexanic RBE were similar, but the exact compositions differed slightly.

**Table 1:** Concentrations of vitamin E congeners (µg/g) in ethanolic and hexanic rice bran extracts (RBE) measured by high-performance liquid chromatography (HPLC) with fluorescence detection.

Vitamin E Congener (µg/g)	Ethanolic RBE	Hexanic RBE
α-Tocopherol	86	112
β-Tocopherol	71	163
γ-Tocopherol	288	163
δ-Tocopherol	93	209
α-Tocotrienol	55	59
β-Tocotrienol	not detected	not detected
γ-Tocotrienol	2226	1791
δ-Tocotrienol	266	591
Total vitamin E	3084	3088

### 2.2. Mitochondrial Function

**Figure 1:**
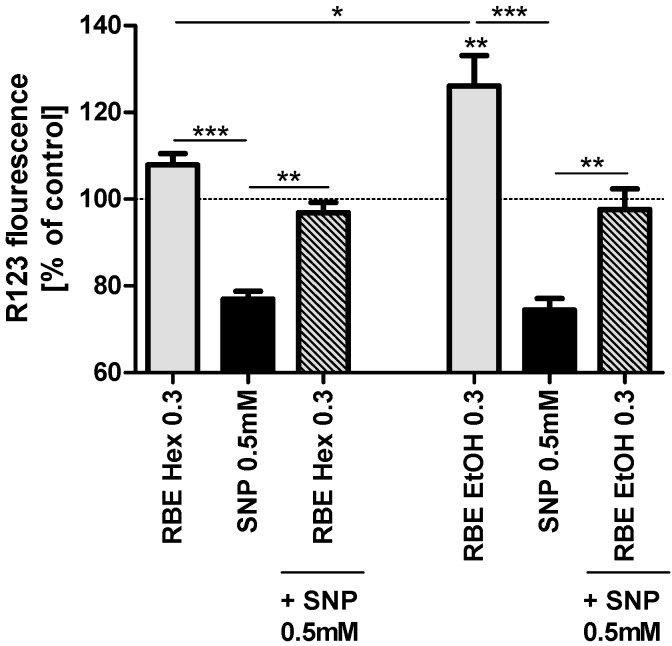
Mitochondrial membrane potential (MMP; gray bars) of PC12 cells after 24-h incubation with 0.3 mg/mL hexanic (RBE Hex) or ethanolic (RBE EtOH) rice bran extract (RBE); MMP after insult with sodium nitroprusside (SNP, 0.5 mM) for 24 h (black bars) and MMP after preincubation of PC12 cells with ethanolic or hexanic RBE for 1 h and insult with SNP (0.5 mM) for 24 h (striped bars); cells treated with cell culture medium served as control for normalization (100%); there were no differences in MMP between medium treated and DMSO or ethanol treated cells (data not shown); *n* = 5–6; mean ± SEM; ANOVA with Tukey’s *post*-test; * *p* < 0.05; ** p < 0.01; *** *p*< 0.001.

To examine the effects of RBE on mitochondrial function we used a concentration of 0.3 mg/mL since this concentration proved to be the most effective concentration in previous experiments [13]. Incubation of PC12 cells for 24 h with the ethanolic RBE (0.3 mg/mL) significantly increased basal mitochondrial membrane potential (MMP, *p* < 0.01), while the hexanic RBE increased basal MMP numerically, but not significantly. Insult of PC12 cells with sodium nitroprusside (SNP, 0.5 mM) significantly decreased MMP to around 80% of that of control cells. Preincubation with either ethanolic or hexanic RBE (0.3 mg/mL) counteracted SNP-induced drop in MMP and resulted in MMP similar to control cells (Figure 1).

A lower concentration of the ethanolic RBE (0.2 mg/mL) also increased MMP (basal and after SNP-stress; *p* < 0.001 for both) similar to the higher concentration of 0.3 mg/mL. The hexanic RBE, on the other hand, did not affect basal MMP or MMP after insult with SNP at lower concentrations of 0.1 and 0.2 mg/mL (data not shown).

**Figure 2:**
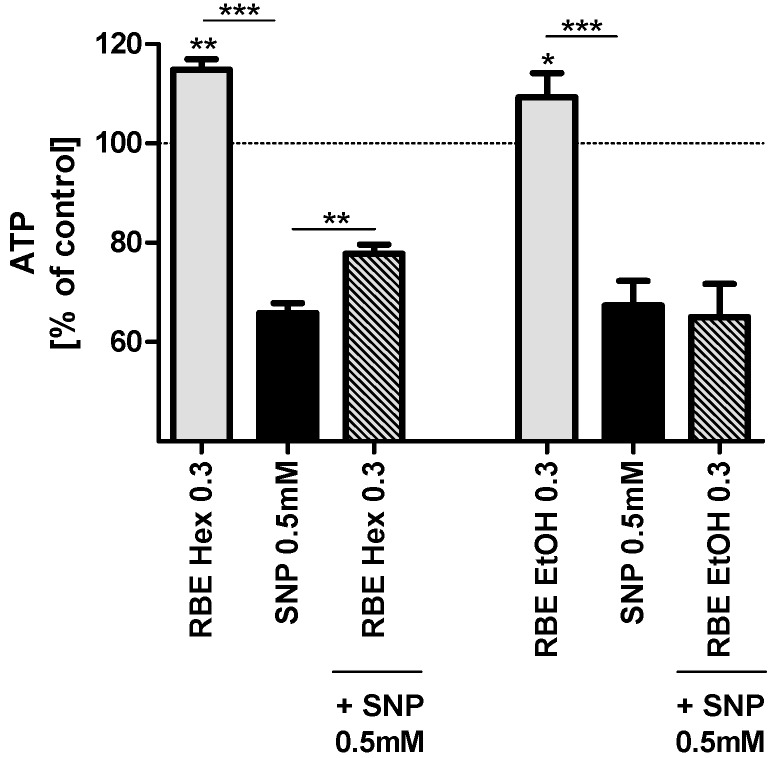
ATP concentrations of PC12 cells after 24-h incubation with 0.3 mg/mL hexanic (RBE Hex) or ethanolic (RBE EtOH) rice bran extract (RBE, grey bars); ATP concentrations after insult with sodium nitroprusside (SNP, 0.5 mM) for 6 h (black bars) and ATP concentrations after preincubation of PC12 cells with ethanolic or hexanic RBE for 1 h and insult with SNP (0.5 mM) for 6 h (striped bars); cells treated with cell culture medium served as control for normalization (100%); there were no differences in ATP between medium treated and DMSO or ethanol treated cells (data not shown); *n* = 6–7; mean ± SEM; ANOVA with Tukey’s *post-*test; * p < 0.05; ** *p* < 0.01; *** *p* < 0.001.

Incubation of PC12 cells with hexanic and ethanolic RBE (0.3 mg/mL) for 24 h significantly increased basal ATP concentrations (*p* < 0.01 and *p* < 0.05; Figure 2). Insult of PC12 cells with SNP (0.5 mM) significantly decreased ATP concentrations to around 70% of control cell concentrations. Preincubation with hexanic RBE (0.3 mg/mL) counteracted the SNP-induced drop in MMP concentrations, while preincubation with ethanolic RBE did not have any significant effect (Figure 2). Smaller concentrations of hexanic or ethanolic RBE did not significantly affect basal ATP concentrations or ATP concentrations after insult of cells with SNP (data not shown).

**Figure 3:**
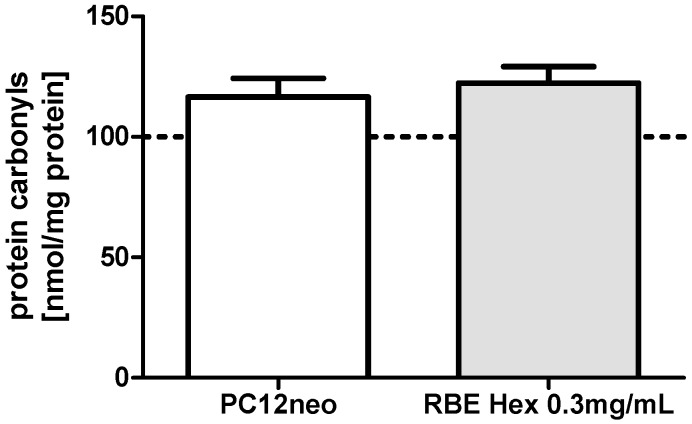
Protein carbonyl concentrations in PC12 cells after 24-h incubation with 0.3 mg/mL hexanic rice bran extract (RBE) or the respective DMSO control; cells treated with cell culture medium served as control for normalization (100%); *n* = 8; mean ± SEM.

Protein carbonyls are formed within cells under conditions of oxidative or nitrosative stress [14]. Protein carbonyl concentrations were not significantly changed by incubation of PC12 cells with hexanic RBE (0.3 mg/mL) for 24 h (see Figure 3). Protein carbonyl concentrations were not determined after incubation of cells with ethanolic RBE.

**Figure 4:**
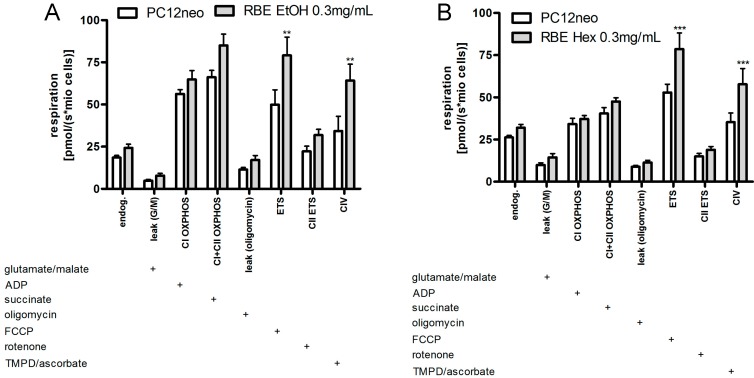
Cell-count normalized respiration of PC12 cells after a 24-h incubation with 0.3 mg/mL ethanolic (RBE EtOH, (**A**)) or hexanic (RBE Hex; (**B**)) rice bran extract (RBE) and the respective solvent control ethanol (**A**) or DMSO (**B**); respiration was measured using an Oxygraph-2k (Oroboros, Austria); the addition of a substance into the Oxygraph chamber is indicated with a plus sign; for a detailed measurement protocol, see Materials and Methods Section; *n* = 9–12; mean ± SEM; ANOVA with Bonferroni *post*-test; ** *p*< 0.01; *** *p* < 0.001.

Incubation of PC12 cells with ethanolic and hexanic RBE (0.3 mg/mL) increased mitochondrial respiration to a similar extent (Figure 4). In detail, incubation with ethanolic RBE significantly increased uncoupled and complex IV respiration (*p* < 0.01). Endogenous respiration, complex I and II as well as OXPHOS respiration were numerically increased following RBE incubation (Figure 4). Incubation with hexanic RBE significantly increased uncoupled and complex IV respiration (*p* < 0.001). Endogenous as well as complex II and OXPHOS respiration were numerically increased (Figure 4).

**Figure 5:**
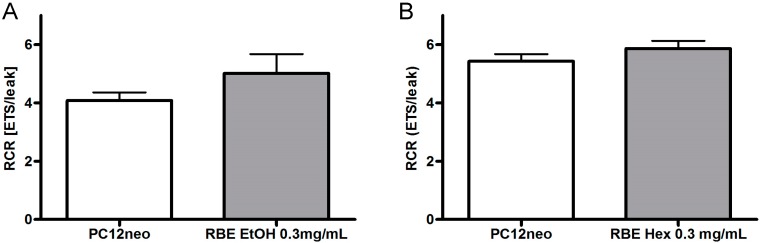
Respiratory control ratio (RCR) of PC12 cells after 24-h incubation with 0.3 mg/mL ethanolic (EtOH, (**A**)) or hexanic (Hex, (**B**)) rice bran extract (RBE) and the respective solvent control ethanol (**A**) or DMSO (**B**); respiration was measured using an Oxygraph-2k; RCR was calculated as ratio between uncoupled respiration and leak respiration after addition of oligomycin; *n* = 9–12; mean ± SEM; ANOVA with Tukey’s *post-*test.

Respiratory control ratio (RCR) was calculated as ratio between OXPHOS respiration and leak respiration after addition of oligomycin [15]. RCR is an indicator for the coupling of the electron transport system [16]. RCR was numerically, though not significantly, increased in PC12 cells after 24-h incubation with ethanolic or hexanic RBE (Figure 5).

**Figure 6:**
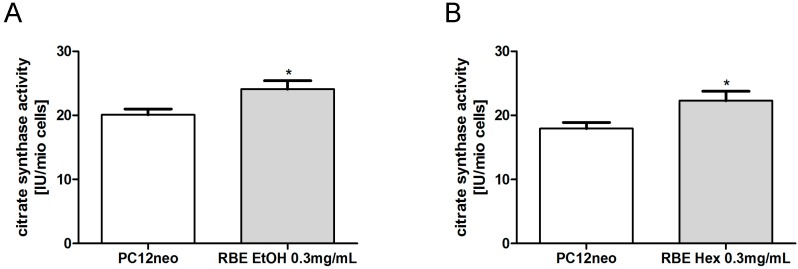
Mitochondrial content marker citrate synthase (CS) activity was significantly increased after 24-h incubation of PC12 cells with ethanolic (RBE EtOH, (**A**)) or hexanic (RBE Hex, (**B**)) rice bran extract (RBE); *n* = 8–12; mean ± SEM; ANOVA with Tukey’s *post-*test; * *p* < 0.05.

Citrate synthase (CS) activity is a common quantitative marker for the content of intact mitochondria [17]. CS activity was significantly increased in PC12 cells after a 24-h incubation with either ethanolic or hexanic RBE (*p* < 0.05; Figure 6).

**Figure 7:**
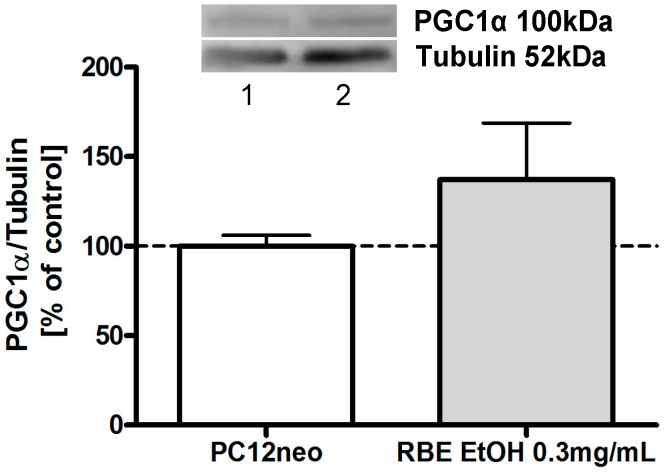
Protein expression of PGC1α, a master regulator of mitochondrial biogenesis, was increased in PC12 cells after a 24-h incubation with ethanolic rice bran extract (RBE); cells incubated with ethanol served as control for normalization; pictures of representative Western blots are depicted in the upper part of the figure (1: EtOH, 2: RBE EtOH 0.3 mg/mL); *n* = 12; mean ± SEM; ANOVA with Tukey’s *post*-test.

Peroxisome proliferator-activated receptor gamma coactivator-1α (PGC1α) is the master regulator of mitochondrial biogenesis and energy expenditure [18]. We have recently shown that incubation of PC12 cells with hexanic RBE (0.3 mg/mL) for 24 h significantly increased PGC1α protein expression [12]. Similarly, 24-h incubation of PC12 cells with ethanolic RBE increased protein expression of PGC1α to around 130%, although this increase did not attain significance (Figure 7).

### 2.3. Single Substances and Fractions of RBE

Both RBE similarly influenced mitochondrial function in PC12 cells by elevating mitochondrial membrane potential, ATP concentrations, mitochondrial respiration and mitochondrial content. We therefore attempted to learn more about the responsible bioactive compound(s) by assessing whether selected components of RBE are solely or mostly responsible for the positive effects of RBE on mitochondrial function. For that purpose, we examined the effects of key components of RBE as pure substances on MMP and ATP concentrations in PC12 cells. Based on tocopherol and tocotrienol concentrations in ethanolic RBE (Table 1), the concentrations of pure substances for cell culture experiments were calculated to represent those present in the extracts. The smaller concentrations tested correspond to the amount of the substance found in 0.3 mg/mL RBE. A five times higher concentration of each substance was tested to reveal potential concentration-dependent effects.

Treatment of PC12 cells with α-tocotrienol, γ-tocotrienol, δ-tocotrienol, α-tocopherol, γ-tocopherol, α-CEHC (CEHC are the end-products of vitamin E metabolism; 100 and 500 nM), γ-CEHC (100 and 500 nM) or ferulic acid (a major phenolic acid of RBE; 4 and 20 µM) for 24 h did not have any effect on basal MMP or MMP after insult with SNP (0.5 mM) for 24 h (see Table 2, part of the data is not shown). Similarly, no effect of the above mentioned substances on basal ATP concentrations after 6 h incubation or ATP concentrations after insult with SNP (0.5 mM) for 6 h could be detected (data not shown).

On the other hand, a 24-h incubation of cells with α-tocopherol (50 nM), δ-tocopherol (70 nM), α-tocotrienol (40 nM) and γ-tocotrienol (1 µM) significantly increased basal ATP concentrations (see Table 2), indicating that these substances contribute to the positive effects of RBE on mitochondrial function.

To confirm our results obtained from the above-described experiments, we additionally examined MMP and ATP concentrations after incubation of PC12 cells with different fractions of rice bran extract. Fraction II is a methanol-soluble fraction comprising around 30% of hexanic RBE. It mostly contains oryzanols and tocotrienols, but no tocopherols; furthermore, it contains plant-derived omega-3 fatty acids, most likely α-linolenic acid. Fraction III is a methanol-insoluble fraction comprising around 60% of hexanic RBE and containing mostly oryzanols. Fraction IV is a polar fraction comprising around 2% of hexanic RBE. Fraction V was extracted from Fraction II and mainly consists of tocotrienols.

**Table 2:** Basal ATP concentrations and mitochondrial membrane potential (MMP) of PC12 cells after 24-h incubation with the indicated pure substances contained in rice bran extract (RBE) and MMP and ATP after preincubation with pure substances for 1 h and subsequent insult with SNP (0.5 mM) for 6 h (ATP) or 24 h (MMP); the solvent control ethanol did not change ATP concentrations significantly; ↑ indicates increased ATP concentrations; -: no significant effect could be detected; n.t.: not tested; *n* = 7–8; ANOVA with Tukey’s *post*-test.

Substance (Concentration)	ATP	ATP + SNP	MMP	MMP + SNP
α-Tocopherol (50 nm)	*p* < 0.05 (↑)	-	-	-
α-Tocopherol (250 nM)	-	-	-	-
γ-Tocopherol (200 nM)	-	-	-	-
γ-Tocopherol (1 µM)	-	n.t.	n.t.	n.t.
δ-Tocopherol (70 nM)	*p* < 0.05 (↑)	n.t.	n.t.	n.t.
δ-Tocopherol (350 nM)	-	n.t.	n.t.	n.t.
α-Tocotrienol (40 nM)	*p* < 0.001 (↑)	-	-	-
α-Tocotrienol (200 nM)	-	n.t.	n.t.	n.t.
γ-Tocotrienol (1 µM)	*p* < 0.05 (↑)	-	-	-
γ-Tocotrienol (5 µM)	-	-	-	-
δ-Tocotrienol (200 nM)	-	-	-	-
δ-Tocotrienol (1 µM)	-	n.t.	n.t.	n.t.

Fraction II increased basal MMP and protected PC12 cells from SNP-induced stress (see Table 3). All other fractions did not have any effects on basal MMP and ATP concentrations or on MMP and ATP concentrations after SNP-insult. Since the available amount of the fractions was limited, we were not able to measure ATP concentrations of PC12 cells after an incubation time of 24 h.

**Table 3:** Basal mitochondrial membrane potential (MMP) and ATP concentrations of PC12 cells after 25-h incubation (MMP) or 7-h incubation (ATP) with fractions extracted from hexanic rice bran extract (RBE),0.3 mg/mL) as well as MMP and ATP concentrations of PC12 cells after preincubation with fractions for 1 h and subsequent insult with SNP (0.5 mM) for 6 h (ATP) or 24 h (MMP); *n* = 4–5; ANOVA with Tukey’s *post*-test; ↑ indicates increased MMP or ATP; -: no significant effect could be detected.

Fraction	MMP	MMP + SNP (0.5 mM)	ATP	ATP + SNP (0.5 mM)
II	*p* < 0.001 (↑)	*p* < 0.001 (↑)	-	-
III	-	-	-	-
IV	-	-	-	-
V	-	-	-	-

### 2.4. Discussion

In this study, vitamin E profiles of ethanolic and hexanic rice bran extracts (RBE) were compared and their effects on mitochondrial function in PC12 cells examined. Furthermore, we aimed to identify the active ingredients or a single component of the extracts that are responsible for the observed beneficial effects on mitochondrial function.

The concentrations of all eight vitamin E congeners were determined in ethanolic and hexanic RBE. Total vitamin E content was similar in both extracts (3 mg/g), with the composition of the congeners varying between the extracts (Table 1). Rice bran contains total vitamin E concentrations of around 0.2 mg/g [19], while RBE have been reported to contain total vitamin E concentrations between 0.36 and 1.1 mg/g [20,21]. Factors influencing total vitamin E content in rice bran and RBE are rice variety, growing area and the solvent and temperature used for extraction [19,20,21]. Accordingly, we conclude that both RBE used in our study have comparatively high total vitamin E contents. According to Chen and coworkers [21], the solvent used for extraction influences total vitamin E content. The use of methanol as extraction solvent led to the highest vitamin E content in the extract; ethyl acetate resulted in medium vitamin E content, and hexane yielded very low vitamin E contents. While the authors conclude that using a high-polarity solvent such as methanol will result in extraction of most phytochemicals, no considerable differences in vitamin E content were observed after using ethanol or hexane as extraction solvents for RBE in the present study.

We then examined the effects of both RBE on mitochondrial function in PC12 cells. Effects of the two extracts were very similar; both extracts increased mitochondrial membrane potential, ATP concentrations, mitochondrial respiration and resistance of cells against nitrosative stress. The underlying mode of action of RBE seems to be increased mitochondrial biogenesis via a PGC1α-dependent mechanism. We recently reported that RBE incubation was able to restore mitochondrial dysfunction in a cell culture model of Alzheimer’s disease (PC12_APPsw_) by increasing mitochondrial content, PGC1α protein concentrations, mitochondrial respiration, ATP production and resistance of cells against nitrosative stress [13]. Similarly, we could show that feeding RBE to young guinea pigs for 30 days improved brain mitochondrial function by increasing mitochondrial content and mitochondrial respiration and by protecting cells from oxidative and nitrosative stress [12]. These results show that the effects of RBE are comparable in cell culture and animal models. The observed effects of ethanolic and hexanic RBE on mitochondrial function of PC12 cells were similar although not exactly identical; probably due to slightly different extract compositions.

Since we were previously able to clarify the mechanism of action of RBE in cell culture and animal models, we now aimed to identify the active ingredient(s) of RBE that is (are) responsible for the beneficial effects on mitochondrial function. For this purpose, we tested the effects of the three known key components of RBE (tocopherols, tocotrienols and γ-oryzanol) as well as of their metabolites α- and γ-carboxyethyl hydroxychroman (CEHC) and ferulic acid on mitochondrial function in PC12 cells. We tested pure substances in concentrations that are comparable to those present in 0.3 mg RBE/mL as well as a five times higher concentration. None of the pure substances showed any effects on basal MMP or on MMP and ATP concentrations after insult with SNP. On the other hand, a 24-h incubation of cells with α-tocopherol (50 nM), δ-tocopherol (70 nM), α-tocotrienol (40 nM and 200 nM) and γ-tocotrienol (1 µM) significantly increased basal ATP concentrations, with α-tocotrienol exhibiting the largest effect. These results indicate that α-tocopherol, δ-tocopherol, γ-tocotrienol and in particular α-tocotrienol contributes to the positive effects of RBE on mitochondrial function. Nevertheless, none of these substances appears to be solely responsible for the effects of RBE on mitochondrial function, since the pure substances did not alter basal MMP and did not protect cells from SNP-induced nitrosative stress.

Over the last few decades, research was mainly focused on α-tocopherol, since this was considered to be the most effective vitamin E compound. Recent research, on the other hand, revealed that tocotrienols might be more effective in protecting neurons and in preventing mitochondrial dysfunction than tocopherols [22,23,24,25,26]. This is in accordance with our observation that α-tocotrienol elevated basal ATP concentrations more effectively than all other vitamin E congeners. Similarly, α-tocotrienol was the only vitamin E form found to accumulate concentration-dependently in guinea pig brains after RBE administration; we therefore hypothesized that it must at least be involved in the concentration-dependent beneficial effects on mitochondrial function that we observed [12]. Accordingly, it stands to reason that α-tocotrienol is one of the bioactive components of RBE.

Like rice bran oil, rice bran extracts most likely contain unsaturated fatty acids such as linolenic acid and isoprenoids such as squalene [27]. ω-3 unsaturated fatty acids have already been reported to protect brain mitochondrial function in guinea pigs [28] and aged mice [29]. Furthermore, ω-3 fatty acids are also known to have various other beneficial health effects [30]. Squalene is especially known for its potential as antioxidant [31]. Recent studies indicated that the ratio of different unsaturated fatty acids is able to influence mitochondrial function [32,33]. According to these results, unsaturated fatty acids in RBE could very well contribute to its beneficial effects on mitochondria. Further experiments will have to be conducted to clarify this matter. Another component of rice bran, which we have so far not examined in our studies, is hydrophilic rice bran saccharide. Rice bran saccharide has been found to have anti-carcinogenic properties [34] but other potential health-beneficial effects of the saccharide have, to our knowledge, not yet been investigated. Nevertheless, it is debatable whether rice bran saccharide is present in our RBEs since hexane and ethanol will not extract hydrophilic compounds. Another possibility for the fact that we could not identify a single component responsible for RBEs beneficial effects on mitochondrial function might be that the mode of action of our extract involves a synergism between different extract components.

To test if a certain group of components is responsible for the beneficial effects of RBE on mitochondrial function, the effects of four different fractions of ethanolic RBE on mitochondrial function have been examined. We tested the effect of the four fractions on basal MMP and ATP concentrations as well as on MMP and ATP concentrations after insult with SNP. Fraction II is a methanol-soluble fraction mainly containing tocotrienols and oryzanols that increased basal MMP and protected MMP from SNP-induced stress. Fraction II did not increase basal ATP concentrations; most likely since the 7-h incubation time was too short and the experiment could not be repeated with a longer incubation time due to limited amounts of RBE fractions. RBE also did not have an effect on basal ATP concentrations after 7-h incubation time. Only after a 24-h incubation time, RBE increased basal ATP concentrations. Therefore, Fraction II has very similar effects on MMP as the whole RBE, but does not affect ATP concentrations. The other three fractions did not significantly affect MMP and ATP concentrations of PC12 cells.

It is likely that tocotrienols and/or γ-oryzanol present in the methanol-insoluble fraction (fraction II) are at least partly responsible for the mitochondria-protective effects of RBE in PC12 cells. Nevertheless, Fraction V (containing tocotrienols and no γ-oryzanol) as well as Fraction III (containing γ-oryzanol and no tocotrienols) did not show any effects on MMP and ATP, which suggests a synergistic effect between at least these two components that enables them to protect mitochondria. Another possibility is the presence of a hitherto unknown component of RBE in Fractions II, III and/or V. A more exact characterization of the extracts and the single fractions will be necessary for the exact identification of all components responsible for the beneficial effects of RBE on mitochondrial function.

## 3. Experimental Section

### 3.1. Chemicals

Unless otherwise stated, chemicals were of highest available purity and purchased from Sigma (St. Louis, MO, USA), Merck (Darmstadt, Germany) or biomol (Hamburg, Germany). Aqueous solutions were prepared with deionized, filtered water (Millipore, Billerica, MA, USA). Heat stabilized Egyptian rice bran extract (RBE) was obtained from IT & M S.A. (Giza, Egypt). After overnight maceration in hexane or ethanol, three successive extraction sessions were applied under reflux at 40 °C. The extraction ratio was 3:1. The extract was evaporated under vacuum at a temperature not exceeding 50 °C. During the experiments, hexanic RBE was dissolved in DMSO and ethanolic RBE was dissolved in ethanol. Fractions of hexanic RBE were prepared by our Egyptian co-operation partners (Prof. Dr. Hesham El-Askary, University of Cairo, Egypt) using preparative HPLC.

### 3.2. Cell Culture

PC12 cells were derived from a pheochromocytoma of the rat adrenal medulla [35]. PC12 cells used in the current study were stably transfected with a plasmid containing a pCMV promoter and a neomycin resistance [36]. Cells were cultured in Dulbecco’s Modified Eagle’s Medium supplemented with 10% heat-inactivated fetal calf serum, 5% heat-inactivated horse serum, penicillin (50 units/mL), streptomycin (50 µg/mL) and G418 (400 µg/mL) in a humidified incubator containing 5% CO_2_.

### 3.3. Mitochondrial Membrane Potential

Two days prior to incubation, cells were seeded into 24-well plates (200,000 cells/well). Cells were incubated with the respective RBE concentrations, pure substances or RBE fractions for 25 h (basal MMP level), or preincubated for 1 h and insulted with SNP (0.5 mM) for 24 h (MMP level after insult). DMSO or ethanol served as the respective solvent controls. MMP was measured using the fluorescence dye rhodamine 123 (R123). Cells were incubated with R123 (0.4 µM) for 15 min in the incubator. Afterwards, the reaction was stopped by adding HBSS buffer (supplemented with Mg^2+^, Ca^2+^ and HEPES; pH 7.4; 37 °C) into the wells. The plate was centrifuged (400× *g*, 5 min), medium was aspirated and new HBSS buffer was added into the wells. MMP was assessed by reading fluorescence at an excitation wavelength of 490 nm and at an emission wavelength of 535 nm (Victor^2^ 1420 multilabel counter, Perkin Elmer, Rodgau-Jügesheim, Germany). The fluorescence in each well was read in four consecutive runs.

### 3.4. ATP Concentrations

Two days prior to incubation, cells were seeded into 96-well plates (20,000 cells/well). Cells were incubated with the respective RBE concentrations, pure substances or RBE fractions for 24 h (basal ATP concentrations), or preincubated for 1 h and insulted with SNP (0.5 mM) for 6 h (ATP concentrations after insult). DMSO or ethanol served as the respective solvent controls. ATP concentrations were assessed using the ViaLight^®^ Plus bioluminescence kit (Lonza, Walkersville, MD, USA), which is based on the production of light from ATP and luciferin in the presence of luciferase. The 96-well plate was removed from the incubator and allowed to cool to room temperature. Thereafter, cells were incubated with lysis buffer for 10 min and with monitoring reagent for an additional 5 min. The emitted light (bioluminescence), which is linearly related to ATP concentrations, was recorded using a luminometer (Victor^2^ 1420 multilabel counter, Perkin Elmer, Rodgau-Jügesheim, Germany).

### 3.5. Mitochondrial Respiration

Cells were incubated with hexanic or ethanolic RBE (0.3 mg/mL) for 24 h; control cells were incubated with the appropriate DMSO or ethanol concentration (0.15%). After incubation, cells were detached from the flask by rinsing with cell culture medium. The cell suspension was centrifuged and cell count was adjusted to one million cells per mL by dilution with MIR05 (a mitochondrial respiration medium developed by Oroboros [37]) containing EGTA (0.5 mM), magnesium dichloride (3 mM), lactobionic acid (60 mM), taurine (20 mM), potassium dihydrogenphosphate (10 mM), HEPES (20 mM), sucrose (110 mM) and essentially fatty acid free bovine serum albumin (1 g/L). To analyze mitochondrial respiration, an Oxygraph-2k system (Oroboros Instruments, Innsbruck, Austria) and the DatLab software version 4.3.2.7 (Oroboros Instruments, Innsbruck, Austria) were used.

To investigate the function of the respiratory system, a complex protocol (elaborated by Prof. Dr. Erich Gnaiger) was applied including different substrates, uncouplers and inhibitors. After adding 2 mL of cell suspension into the two chambers of the Oxygraph-2k, chambers were closed and respiration stabilized (endogenous respiration). Afterwards, the plasma membrane of the cells was permeabilized with digitonin (1 µg/10^6^ cells), leaving mitochondrial outer and inner membrane intact (permeabilized). The capacity of oxidative phosphorylation was determined with complex I related substrates (CI) glutamate (10 mM), malate (2 mM) and ADP (2 mM) followed by addition of succinate (10 mM; OXPHOS). Leak respiration after addition of glutamate/malate is labeled leak (G/M) and corresponds to state 4 respiration, further addition of ADP induces state 3 respiration. Oligomycin (2 µg/mL) addition allowed measurement of the second leak respiration state leak (omy). Subsequently, uncoupling (ETS) was achieved by addition of carbonyl cyanide *p*-(trifluoromethoxy) phenylhydrazone (FCCP, injected stepwise up to 2.5 µM). Complex II respiration in the non-coupled state (CII_ETS_) was monitored after adding rotenone (0.5 µM) into the chambers. Residual oxygen consumption (ROX), oxygen consumption caused by enzymes which do not belong to the electron transfer system, was determined after inhibition of complex III by addition of antimycin A (2.5 µM) and was subtracted from all respiratory parameters. COX activity (CIV) was measured after ROX determination by applying 0.5 mM tetramethyl-phenylenediamine (TMPD) as an artificial substrate of complex IV and 2 mM ascorbate to keep TMPD in the reduced state. Autoxidation rate was determined after addition of sodium azide (≥100 mM) and COX respiration was additionally corrected for autoxidation.

### 3.6. Citrate Synthase Activity

A subsample of the cell suspension used for mitochondrial respiration measurements was immediately frozen in liquid nitrogen for citrate synthase activity determination. After thawing, a reaction medium containing 0.1 mM DTNB, 0.5 mM oxaloacetate, 50 µM EDTA, 0.31 mM acetyl coenzyme A, 5 mM triethanolamine hydrochloride and 0.1 M Tris-HCl was mixed and preheated for 5 min at 30 °C. Subsequently, 200 µL of mitochondria were added to the reaction medium and citrate synthase (CS) activity was assessed spectrophotometrically at 412 nm [38,39]. Measurements were performed in triplicates.

### 3.7. Western Blot Analysis

Cells were homogenized in lysis buffer (containing 1 mM EDTA, 0.5% Triton X-100, 5 mM NaF, 6 M urea, 25 mM sodium pyrophosphate, 1 mM sodium orthovanadate, 0.5% sodium deoxycholate, 0.5% sodium dodecyl sulfate, aprotinin (1.7 mg/mL), leupeptin (5 mg/mL), pepstatin (5 mg/mL) and PMSF (100 mM)).

Samples (containing 10–20 µg protein) were mixed with Tris/glycine reducing buffer and denaturing loading buffer (Invitrogen™, Carlsbad, CA, USA) before they were loaded and electrophoresed on NuPAGE™ 4%–12% Bis-Tris Gels (Invitrogen™, Darmstadt, Germany). Gels were transferred to PVDF membranes (Amersham Biosciences, Chalfont St Giles, UK), incubated with the respective primary antibodies (PGC1α (ab106814), tubulin (ab6160), Abcam, Cambridge, UK) and secondary antibodies (Calbiochem, Darmstadt, Germany) conjugated to horseradish peroxidase and processed for visualization by ECLplus™ Reagent (Amersham Biosciences). Tubulin (Abcam, Cambridge, UK) served as loading control. Two replicates of each measurement were carried out; band analysis was performed using BioRad’s Quantity One software (BioRad, Hercules, CA, USA).

### 3.8. HPLC Analyses of Vitamin E Congeners

Tocopherols and tocotrienols in the RBE were quantified by a validated HPLC method with fluorescence detection as previously described [40].

### 3.9. Statistics

All data are presented as mean ± SEM. Statistical analysis was performed by applying one-way ANOVA with Tukey’s or Bonferroni post-tests (Prism 5.0, GraphPad Software, San Diego, CA, USA). A *p* value of <0.05 was considered statistically significant.

## 4. Conclusions

Altogether, our results indicate similar vitamin E profiles and similar effects of hexanic and ethanolic rice bran extracts on mitochondrial function in PC12 cells. Therefore, ethanolic RBE, which shows strong mitochondria-protective effects in cultured cells, might be suitable for the prevention of age-related neurodegeneration and AD and should be further investigated in appropriate animal and human trials. Ultimately, ethanolic RBE could be a functional ingredient for nutraceuticals that could easily be included in daily eating habits.
